# Non-literal language and semantic dementia

**DOI:** 10.1590/S1980-57642013DN74000011

**Published:** 2013

**Authors:** Mariana Ribeiro Hur, Leonardo Caixeta

**Affiliations:** 1 Speech and Hearing Therapist, Master’s Student in Health Sciences at the Federal University of Goiás - UFG, Goiânia GO, Brazil.; 2 Medical Doctor, PhD, Assistant Professor of Neuropsychiatry of the School of Medicine of the Federal University of Goiás, UFG, Coordinator of the Cognitive and Behavioral Neurology Unit of the Clinicas Hospital of the UFG, Goiânia GO, Brazil.

**Keywords:** frontotemporal lobar degeneration, language, language tests, semantic dementia

## Abstract

Semantic dementia is characterized by fluent, phonologically adequate speech with various anomias and semantic paraphasias. Performance on semantic tasks is well documented in these patients, although little is known regarding performance on more complex language tasks, such as those involving non-literal language (interpretation of metaphors and proverbs and recognition of irony).

**OBJECTIVE:**

To report the investigation of non-literal language in cases of semantic dementia.

**METHODS:**

Two cases of semantic dementia were investigated for the presence of deficits in non-literal language abilities using the screening test for Alzheimer's disease with proverbs, metaphor test and irony test.

**RESULTS:**

Both patients were found to have low performance on the tests applied, particularly for interpretation of proverbs.

**CONCLUSION:**

This poor performance was attributed largely to the characteristic semantic changes of the disease, but some frontal symptoms inherent to other forms of frontotemporal lobar degeneration were also observed which interfered in the testing, such as negativism, reduced attention span, concretism and perseverations.

## Abstract (pt)

A demência semântica é caracterizada por fala fluente e adequada fonologicamente e com diversas anomias e parafasias semânticas. O desempenho em tarefas semânticas é bem documentado nestes pacientes, porém pouco se sabe acerca do desempenho em tarefas linguísticas mais complexas, como naquelas que envolvem linguagem não literal (interpretação de metáforas e provérbios e reconhecimento de ironias).

**OBJETIVO:**

Relatar a investigação da linguagem não-literal em casos de demência semântica.

**MÉTODOS:**

Dois casos de demência semântica foram investigados para a presença de déficits em habilidades de linguagem não-literal, usando o teste de triagem para a doença de Alzheimer com provérbios, teste de metáforas e teste de ironia.

**RESULTADOS:**

Verificou-se que ambas as pacientes apresentaram baixo desempenho nos testes aplicados, principalmente na interpretação de provérbios.

**CONCLUSÃO:**

O baixo desempenho foi atribuído especialmente às alterações semânticas características da doença, porém também foram observados sintomas frontais característicos de outras formas de degeneração lobar frontotemporal que interferiram na testagem como negativismo, redução do fôlego atencional, concretismo e perseverações.

## INTRODUCTION

Semantic dementia (SD) is one of the three subtypes of frontotemporal lobar degeneration (FTLD) and is characterized by marked atrophy of the temporal lobes bilaterally, more specifically of the anterior temporal neocortex.^1^ This temporal cortex atrophy produces a distinct language profile.^2^

SD patients produce fluent and phonetically adequate speech which is meaningless and exhibits numerous semantic paraphasias and anomias. Thus, loss of meaning is the central characteristic of SD and affects both oral output and comprehension.^3-6^

Recently, a number of studies have been published investigating performance of patients with dementia on non-literal language tasks. Based on these studies, it is known that patients with FTLD of the frontotemporal dementia (FTD) type display poor performance, mainly owing to difficulties in abstraction, attention, minimal effort, and perseverations. However, little is known about the performance of SD patients on these tasks, since studies carried out in this field tend to be centered on changes in the semantic sphere.

Therefore, a case series of two patients with semantic dementia is reported in which possible deficits in non-literal language abilities are investigated by tests assessing the main skills in the use of non-literal language: interpreting of proverbs and metaphors, and recognition of irony.

## METHODS

Non-literal language was investigated in two cases of semantic dementia.

**Patient 1.** A 60-year-old housewife, functionally illiterate, She experienced onset of progressive condition two years earlier with difficulty accessing the lexicon, loss of meaning of less common words and repetitive speech. Progressive changes in personality rendered her apathetic, whereas she was hitherto highly active, sociable and communicative.

Around one year ago, she had a significant reduction in verbal output and the patient became increasingly mute and showed reduced fluency of speech. A substantial worsening of anomia and difficulty understanding the meaning of common words was also observed.

Currently, the patient displays neglect of personal hygiene, placidity, hyperphagia and social inadequacy.

**Patient 2.** A 68-year-old woman, former University teacher, holding PhD in Linguistics. She experienced onset of progressive condition four years earlier with initial symptoms of anomias and difficulties understanding certain words. Personality changes occurred, where her previous shy and quiet nature gave way to a more outgoing personality. She used to be a classical pianist but lost interest in playing and attending concerts.

As the disease evolved, auditory comprehension and anomias worsened with the emergence of a number of semantic paraphasias.

Currently, the patient displays disinhibition, puerile socially inadequate behavior and hyperphagia.

Both patients were diagnosed with semantic dementia according to the criteria proposed by Neary et al (1998).^6^

The procedures described below were approved by the research ethics committee of the Federal University of Goiás - UFG and the guardians of the patients signed a free and informed consent form.

## Imaging exams

Magnetic resonance imaging (MRI) – 

**Figure 1:**
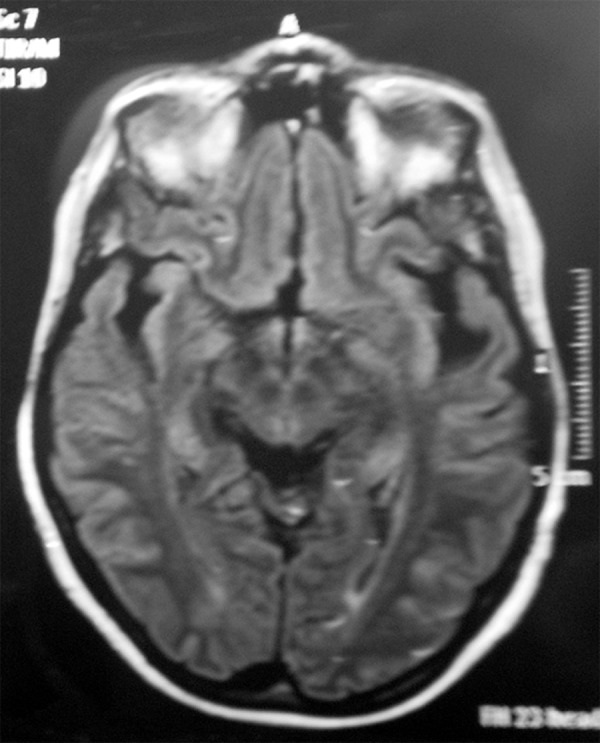
Cranial magnetic resonance image (axial T2) showing bilateral asymmetric temporal atrophy (greater to the left) also involving the hippocampus.

Patient 1: Asymmetric bilateral temporal atrophy, greater to the left, and hippocampus atrophy more evident to the left. Patient 2: Figure 1.

**Figure 2:**
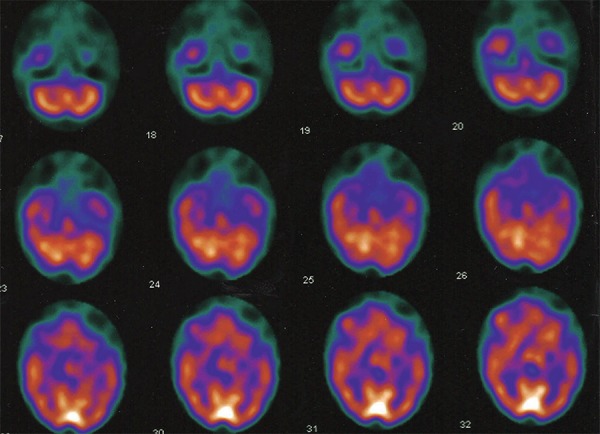
Brain SPECT (axial cuts) showing moderate asymmetric hypoperfusion in temporal lobes (L > R).

*SPECT – *Patient 1: Figure 2. Patient 2: Moderate hypoperfusion in frontal and temporal, more pronounced on the left.

**Non-literal language tests.** For the evaluation of non-literal language we used the Screening test for Alzheimer's disease with proverbs (STADP),^7^ Metaphor test – Test of language competence^8^ and Irony test.^9^

## RESULTS

**Screening test for Alzheimer's disease with proverbs (STADP).^7^** On this test, patient 1 failed to correctly complete any of the 6 popular Brazilian proverbs presented. She questioned less common words, repeating them several times. Regarding interpretation, the patient did not use abstraction when answering the proverbs.

Patient 2 had similar results, being unable to complete or interpret any of the proverbs presented.

**Metaphor test – Test of language competence.^8^** On this test, patient 1 failed to correctly answer any of the 12 metaphors presented. The patient refused to answer for 5 of the sentences and either questioned unfamiliar words again or gave the literal meaning of one of the words of the phrase in the other 7 sentences.

Patient 2 however, correctly interpreted 4 of the metaphors presented. She questioned the meaning of some more unusual words and answered some questions in a concrete and perseverative manner.

**Irony test.^9^** On this test, patient 1 correctly answered for 4 out of the 18 tales presented. The correct answers were achieved when the patients was paying attention to the reading of the tale. On the other tales, the patients answered impulsively or introduced content from their personal life and did not fulfill the purpose of the testing.

Patient 2 achieved 10 correct answers. The patient paid attention throughout the application of the test and benefited from the fact the test elicited a yes/no answer, as opposed to requiring the use of abstraction.

**Qualitative analysis.** During testing, patient 1 had little interaction with the examiner, maintaining a stance of refusal to cooperate and persistent negativity.

With regard to the test, a reduced attention span was a notable factor. A marked display of minimal effort was also evident, whereby the patient repeatedly answered "don't know" so she did not have to answer the question posed. The patient also consistently gave impulsive and spontaneous answers.

On the other hand, patient 2 interacted well with the examiner and cooperated during the testing, despite some puerile behavior at certain points.

A marked difficulty using abstraction was noted, where the patient tended to give concrete answers. The patients also exhibited perseveration at some points.

## DISCUSSION

In the more common subtype of FTLD, frontotemporal dementia, we found significant language deficits, such as progressive reduction in discourse, echolalia, perseveration and stereotyped use of language. Owing to impaired executive functions, changes in social use of language was observed as well as in dealing with non-verbal language signs or with tasks that called for greater abstraction.^ 10-12^

In semantic dementia however, it is uncommon to see executive function deficits that cause problems on more complex language tasks. In this case, it is believed that impairment in semantic memory and semantic comprehension of language can affect the performance of these patients on tasks requiring manipulation of the language material on different levels, including semantic and pragmatic, among others.

Regarding interpretation of proverbs, Báez et al. (2009)^13^ reported that the main aspect governing this interpretation is the familiarity of the patient with the proverb tested, and for this reason we selected a test in which the patients had to initially complete the proverb. The patients assessed failed to properly complete any of the proverbs presented. Given the case of semantic dementia involved, one may hypothesize that the difficulty on this task stems from the impaired ability to attribute meaning to the language structures (words and phrases) and from the significant changes in semantic memory inherent to this pathology.^14^ The difficulty attributing meaning to the words contained in the proverbs appears to hamper their subsequent interpretation.

For the metaphor tests, the patients assessed exhibited extreme difficulties on the task. In a study published in 2009, Schmidt & Seger^15^ reported that the ability to interpret metaphors chiefly involves the left temporal lobe, the neuroanatomical area most affected in semantic dementia. The patients assessed in the present study exhibited structural and functional changes in the left temporal lobe, as can be seen in Figures 1 and 2.

Overall, the two patients assessed in this case series had problems identifying irony, despite successfully recognizing some instances. As outlined earlier, the patients had significant problems attributing meaning to some words (in general less common words), which appears to have interfered with the interpretation of the tale as a whole, in that the patients struggled to attribute meaning to these words. In addition, patients seemed to be unable to correctly attribute mental states to the characters, since even in the tales on which there were no apparent semantic difficulties, they did not manage to detect irony.

Rankin et al.^16^ performed a study on the comprehension of irony in a population with a variety of different types of neurodegenerative diseases, including semantic dementia. In the study, the patients with semantic dementia were the only group of subjects to encounter difficulties recognizing irony. The authors attributed this poor performance to those areas most compromised in SD, namely, the anterior areas of the temporal lobes, which were shown to be the neuroanatomical areas most recruited on this task.

Besides the semantic changes which clearly contributed to the low performance observed, the presence of behavioral changes and of frontal executive symptoms such as minimal effort, lack of attention and perseverance appear to have also exerted an influence on this performance. Rabinovici & Miller^17^ reported that behavioral symptoms may be present from the initial stages of this pathology and may affect the patient's social interaction. Furthermore, with disease progression, deficits in executive functions may develop owing to increased atrophy which begins to involve frontal areas. We noted that patient 2 had areas of hypoperfusion in the frontal lobes, disclosed by the functional neuroimaging exam.

Thus, the analysis of this series of semantic dementia revealed that the patients assessed had significant difficulty regarding all of the non-literal language abilities tested, as a result of the characteristic semantic impairments of this form of dementia, and owing to the behavioral and executive function deficits, features of frontotemporal dementia which can be present in patients with semantic dementia.
